# Editorial: Rare diseases research and diagnosis in low- and middle-income countries

**DOI:** 10.3389/fgene.2025.1675361

**Published:** 2025-08-20

**Authors:** Claudia Gonzaga-Jauregui, Ferran Casals, Vajira H. W. Dissanayake

**Affiliations:** ^1^ International Laboratory for Human Genome Research, Laboratorio Internacional de Investigación sobre el Genoma Humano, Universidad Nacional Autónoma de México, UNAM, Querérato, Mexico; ^2^ Departament de Genètica, Microbiologia i Estadística, Facultat de Biologia, Universitat de Barcelona, Barcelona, Spain; ^3^ Institut de Biomedicina de la Universitat de Barcelona (IBUB), Universitat de Barcelona, Barcelona, Spain; ^4^ Instituto de Salud Carlos III, Centro de Investigación Biomédica en Red de Enfermedades Raras (CIBERER), Barcelona, Spain; ^5^ Institut de Recerca Sant Joan de Déu, Barcelona, Spain; ^6^ Department of Anatomy, Genetics and Biomedical Informatics, Faculty of Medicine, University of Colombo, Colombo, Sri Lanka

**Keywords:** rare diseases, genetics, genomics, low and middle income countries, molecular diagnostics, next-generation sequencing (NGS), equity, research

Rare diseases (RDs) encompass more than 7,000 described disorders characterized by a low prevalence in the general population. Collectively, these disorders affect between 6% and 8% of the world population. The majority of RDs involve an underlying genetic component, and more than 6,000 conditions have been linked to a known molecular cause. In the last 15 years, the adoption of human genomic sequencing has enabled the more efficient and accurate diagnosis and research of rare genetic disorders. Genomic sequencing has become a first-tier diagnostic test for many patients with congenital syndromes and suspected genetic disorders in high-income countries, as well as an effective method for the study of undiagnosed and novel genetic disorders in the research arena. The implementation of massive parallel sequencing technologies, in the form of candidate gene panels, exome, or whole genome sequencing, has dramatically changed the diagnosis and research of rare diseases in high-income countries. In contrast, the reality in low- and middle-income countries (LMICs) is strikingly different, where disparities in access to these technologies exist. The high cost of genomic sequencing and other genetic analysis technologies remains a limiting factor for the implementation of these methods for diagnosis and research of rare diseases in resource limited settings. The study of rare genetic diseases in LMICs remains significantly underrepresented compared to large-scale genomic studies conducted in high-income countries. Despite some being carried out under resource-constrained conditions, these efforts often receive limited visibility in the scientific literature. As a result, there is a disproportionate lack of data from LMICs, which restricts the global understanding of the genetic and phenotypic diversity of RDs and contributes to the persistent underrepresentation of non-European populations in genomic research.

In this Research Topic focused on *Rare Diseases Research and Diagnosis in Low- and Middle-Income Countries* we have captured some of the efforts undergoing in LMICs to bridge the gap of access to diagnosis and research investigating RDs. Clinicians and researchers from 20 different LMICs contributed work that spanned interesting case reports for rare diseases in diverse populations to efforts for the implementation of genomic sequencing programs in resource limited settings. Case reports in this Research Topic report a first diagnosed patient with Fabry disease in North Macedonia (Gjorgjievski et al.), a patient from Colombia with vascular Ehlers–Danlos syndrome (Valencia-Cifuentes, et al.), a Malaysian patient with *ESCO2* associated disorder (Tae et al.), and patients with mitochondrial DNA depletion syndrome, primary ciliary dyskinesia, and spastic paraplegia due to rare novel variants in *RRM2B*, *RSPH4A* and *WASHC5*, respectively, in China (Wang et al.; Shen et al.; Gao et al.).

Larger implementation efforts reported here include those from Yilmaz et al. describing the results of a pilot study for the implementation of rapid genome sequencing in a hospital setting in Turkey to provide accurate and timely diagnoses for critically ill patients in neonatal and pediatric intensive care units. Zharmakhanova et al. present a study protocol outlining the design of an upcoming study to assess the prevalence of inborn errors of metabolism among children in Kazakhstan using the LC-MS/MS method. Other efforts have been directed at the diagnosis of some specific rare diseases, such as syndromic deafness, mitochondrial diseases, and monogenic diabetes in Tunisia (Mkaouar et al.; Gouiza et al.; Kheriji et al.), neurofibromatosis type 1 in South Africa (Mudau et al.), or syndromic short stature in patients from China (Sun et al.).

Importantly, biases in the diagnosis of certain conditions in some populations versus others can delay diagnosis and appropriate treatment and management of patients with RDs. Such is the case of cystic fibrosis in patients from North African and other non-European populations where prevalence and genetic epidemiology information is scarce and clinical overlap with other conditions complicate the diagnosis of this condition in the absence of molecular and genomic testing technologies as reviewed by Makhzen et al. Similarly, Campbell et al. evaluated the utility of singleton clinical exome sequencing for the diagnosis of South African infants from two state hospitals, achieving an overall 22% diagnostic rate in a population where the lack of representation presents a challenge for the accurate interpretation of genomic variants. The analysis of underexplored populations is also of great interest for identifying new variants not previously reported, as, for example, reported by Abdulkareem et al. in the analysis of the genetic causes of epilepsy in consanguineous families of Pakistani origin.

Molecular investigations of patients with suspected genetic disorders are important to determine the exact molecular causes of RDs; however in many LMICs, patients living with RDs rarely have access to molecular testing. Even in cases of well-characterized genetic disorders, it is most relevant to perform molecular testing, as shown by Brito et al. through their study of patients with a clinical diagnosis of Rett syndrome where 42.8% were found to have pathogenic variants in *MECP2*, whereas 28.5% of patients had pathogenic variants in other genes associated with neurodevelopmental disorders. Additional articles in this Research Topic focused on the characterization of the clinical and variant spectrum of certain RDs, such as Kabuki syndrome (Boniel et al.), and merosin-deficient congenital muscular dystrophy type 1a in Vietnamese patients (Khanh Tran et al.). Other reports explore more in depth the mechanisms of certain rare diseases, such as dysregulation of endoplasmic reticulum stress-related genes in dilated cardiomyopathy (Chen et al.), abnormal expression of genes in patients with *CTCF* neurodevelopmental disorder (Tan et al.), or the aberrant splicing that results in an abnormal protein in a patient with Stickler syndrome type I (Gong et al.). Studies of variation causing rare diseases can have implications for families, enabling genetic counseling and testing for family planning as shown by Wu et al. with the identification of genetic variants for citrullinemia type-1 and by Tian et al. studying a novel splicing variant associated with hereditary spherocytosis for preimplantation genetic testing in a Chinese family.

Other reviews included in this Research Topic cover the advantages and challenges of incorporating CNV analyses from exome data to improve the yield of exome sequencing in resource-constrained settings (Louw et al.) where the availability and affordability of whole genome sequencing is still lagging. Additionally, Giugliani et al. review the current landscape and challenges of newborn screening programs in Latin America. Despite demonstrated to be a highly-effective public health strategy, newborn screening is still not broadly implemented in Latin America and other LMICs in the world. Early and accurate detection of rare diseases is fundamental to reduce mortality and morbidity associated with RDs and ensure quality of life for patients.

In summary, the wide array of papers published as part of this Research Topic highlight that even in resource constrained settings, molecular genetic testing, including genomic sequencing, for rare disorders is extremely valuable for diagnosis, research, management and counseling of patients and families living with RDs. Efforts should continue and increase to make these technologies more broadly available and affordable to be successfully implemented in LMICs. This Research Topic adds to the rapidly expanding knowledge coming from historically underrepresented populations and countries and should be welcomed by all those in the field who wish to see genetic and genomic data from global populations represented in international genomic databases.

